# *Toxoplasma gondii* in rodents and shrews in Armenia, Transcaucasia

**DOI:** 10.1016/j.ijppaw.2024.100987

**Published:** 2024-09-11

**Authors:** Sargis A. Aghayan, Manan V. Asikyan, Oleg Shcherbakov, Astghik Ghazaryan, Tigran Hayrapetyan, Alexander Malkhasyan, Hasmik Gevorgyan, Arseny Makarikov, Svetlana Kornienko, Ahmad Daryani

**Affiliations:** a Laboratory of Molecular Parasitology, Scientific Center of Zoology and Hydroecology, NAS RA, 7 P. Sevak st., Yerevan, 0014, Armenia; b Chair of Zoology, Yerevan State University, 1 Alek Manukyan St, Yerevan, 0025, Armenia; c Research Center of Veterinary and Sanitary Expertise, Armenian National Agrarian University, 74 Teryan St, Yerevan, 0025, Armenia; d WWF-Armenia, 11/1 Proshyan Str., Yerevan, 0019, Armenia; e Laboratory of Parasitology, Institute of Systematics and Ecology of Animals SB RAS, Ulitsa Frunze, 11, Novosibirsk, 630091, Novosibirsk Oblast, Russia; f Toxoplasmosis Research Center, Communicable Diseases Institute, Mazandaran University of Medical Sciences, Mazandaran Province, Sari, North Ring, H27P+84G, Iran

## Abstract

*Toxoplasma gondii* infections in small mammals are important because they serve as source of infection for the felids who excrete environmentally resistant oocysts in their feces. Here, the authors sought evidence for *T. gondii* infection in shrews and rodents in Armenia for the first time. *Toxoplasma gondii* DNA was detected in tissues of trapped animals using a specific PCR targeting gene with a non-coding fragment length of 529 bp. *Toxoplasma gondii* DNA was detected in 15 out of 137 (10.9%) samples from small mammals from 6 different localities of Armenia for the first time.

## Highlights

• None of the representatives of the order Eulipotyphla were infected by *T. gondii.*
• The prevalence of infection among rodents was 13.4%, with the highest on in *Dryomis nitedula*.
• A difference in *T. gondii* prevalence was found between male and female rodents, with females carrying no infection.
• There was no significant influence of host age and locality on the prevalence of *T. gondii* in rodents.

## Graphical abstract

**Figure:**
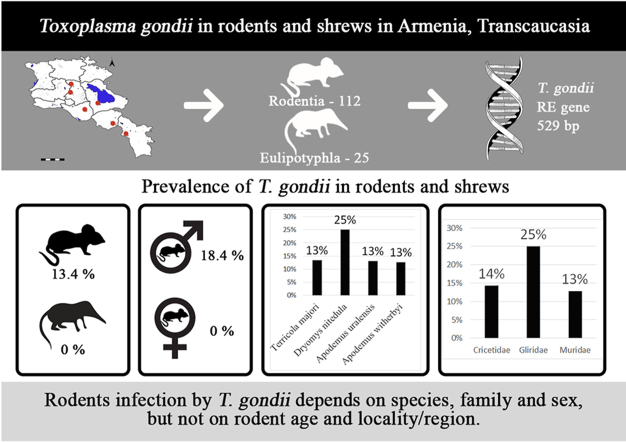

*Toxoplasma gondii* infection is a worldwide zoonosis. It infects various species of mammals, birds, and humans, leading to toxoplasmosis, which can range from asymptomatic to severe and occasionally fatal disease (Ferguson, 2009). Humans become post-natally infected with *T. gondii* by ingesting food and water contaminated with oocysts excreted in the feces of the definitive hosts, felids or by eating infected meat. Cats themselves become infected with *T. gondii* by preying on infected small mammals and birds (Dubey et al., 2021). Although rodents and small mammals have been found infected with *T. gondii* worldwide (Galeh et al., 2020, 2022), we are not aware of any reports of *T. gondii* infections in small mammals in Armenia.

The goal of our study was to investigate the rate of *T. gondii* DNA in rodents and insectivores in Armenia and explore factors influencing prevalence and distribution.

Field sampling was conducted from June to September 2018 in 6 different localities in Armenia (Fig. 1).

**Fig. 1:**
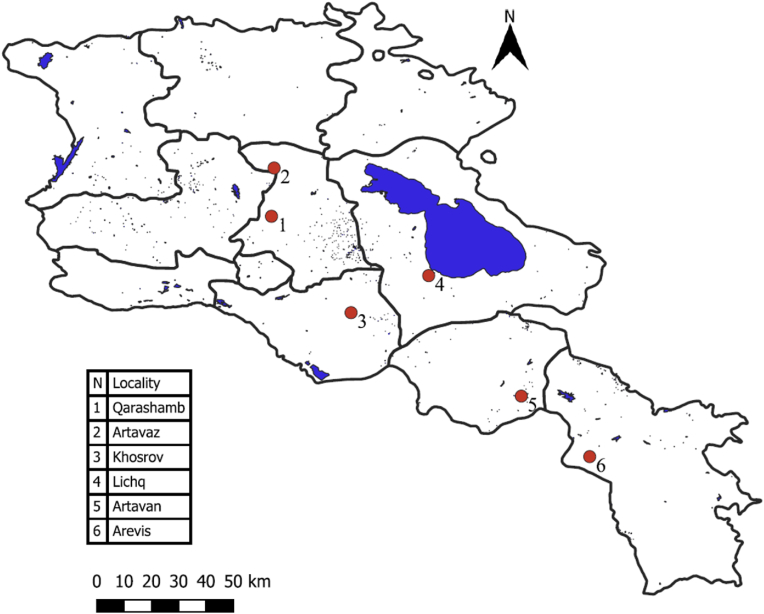
Sampling localities in Armenia: The numbers on the map correspond to the numbers in the table with names of locations.

Animals were captured using Sherman traps placed in forest localities at 7 p.m., with the animals collection conducted the following day at 7 a.m. Initially, the morphological identification of the captured small mammals was carried out. Species names were referenced from The Integrated Taxonomic Information System (ITIS Global) (“Integrated Taxonomic Information System,” n.d.) and IUCN (IUCNRedList, n.d.). Blood samples from the captured animals were collected and preserved for future examination in 96% ethanol (approximately 50% blood and 50% ethanol). In total, 137 samples of 14 species were collected (Table 1).

**Table 1:** Number and prevalence (%) of *T. gondii* in studied rodents and shrews.

Species	Number of samples	Prevalence n (%)
***Rodentia***		
Gliridae		
*Dryomys nitedula* (Pallas, 1778)	9	3 (33)
Muridae		
*Apodemus uralensis* (Pallas, 1811)	46	6 (13)
*Apodemus witherbyi* (Thomas, 1902)	32	4 (13)
*Cricetidae*		
*Mesocricetus brandti* (Nehring, 1898)	2	0 (0)
*Microtus arvalis* (Pallas, 1778)	1	0 (0)
*M. daghestanicus* (Shidlovsky, 1919)	4	0 (0)
*M. majori* (Thomas, 1906)	17	2 (12)
*Chionomys nivalis* (Martins, 1842)	1	0 (0)
***Eulipotyphla***		
*Soricidae*		
*Crocidura leucodon* (Hermann, 1780)	4	0 (0)
*C. suaveolens* (Pallas, 1811)	7	0 (0)
*Neomys teres* (Miller, 1908)	3	0 (0)
*Sorex satunini* (Ognev, 1922)	5	0 (0)
*S. raddei* (Satunin, 1895)	1	0 (0)
*S. volnuchini* (Ognev, 1921)	5	0 (0)
TOTAL	137	

DNA extraction from the collected blood samples were performed using corresponding protocols of Extran 2 DNA extraction KIT (EX-511-100, Synthol, Russia).

*Toxoplasma gondii* was identified using specific primers derived from RE gene with a non-coding fragment length of 529 bp (*Toxo-4:* CGCTGCAGGGAGGAAGACGAAAGTTG and *Toxo-5:* CGCTGCAGACACAGTGCATCTGGATT). The PCR reaction with a final volume of 25 μl, containing 12.5 μl master mix 1x, 0.5 μl of each primer (0.2 μM), 5 μl of DNA template and 6.5 μl deionized water․ Conditions for PCR reaction were as follows: initial hot start at 95 °C for 5 min, 35 cycles of each consisting of denaturation for 30 s at 94 °C, annealing for 30 s at 53 °C, elongation for 40 s at 72 °C and a final extension step at 72 °C for 5 min. For visual detection by ultraviolet transillumination, we used 1.5% agarose gel electrophoresis with SYBR® Green stain (Homan et al., 2000; Hosseini et al., 2020).

A total of 15 of 137 (10.9%) blood samples from rodents were PCR positive, none of the 25 representatives of the order Eulipotyphla were infected by *T. gondii* (Table 1). The overall prevalence of infection among rodents is 13.4%.

Among rodent species, *Dryomis nitedula*, the sole representative of the family Gliridae in our collection, had the highest prevalence of *T. gondii* DNA (Table 1). Among different age groups of rodents, adults were the most infected (13/79: 16.5%) followed by juveniles (1/12: 8.3%) and sub-adults (1/16: 6.3%). However, the difference was not statistically significant.

Statistically significant difference between prevalence of *T. gondii* DNA in males and females was found. None of female rodents were positive to *T. gondii*, while 16 out of 87 males (18.4%) were positive to the agent (Chi-square value: 5.045, P-value: 0.02470). However, our observations were limited to DNA in blood; serological and bioassay might provide more definitive result.

The highest prevalence was recorded in Karashamb, Kotayk region (Table 2). The rodents from the regions of Vayots Dzor and Syunik regions were free of *T. gondii* infection, but the sample size was small and uneven.

**Table 2:** Prevalence of *T. gondii* infection in rodents by localities.

Regions	Locality	Number of samples	Prevalence (%)	Altitutde
Vayots Dzor	Artavan	7	0 (0)	1850
Gegharkunik	Lichk	16	3 (19)	1905
Syunik	Arevis	19	0 (0)	1890
Ararat	Khosrov	18	2 (11)	1350
Kotayk	Karashamb	4	1 (25)	1455
Kotayk	Artavaz	48	7 (17)	1830

Future research with larger sample sizes is essential for a more detailed description of diseases carried by different rodent species in Armenia, considering also the spacial analyzes in study design.

## CRediT authorship contribution statement

**Sargis A. Aghayan:** Writing – review & editing, Writing – original draft, Supervision, Project administration, Investigation, Funding acquisition, Data curation, Conceptualization. **Manan V. Asikyan:** Writing – review & editing, Investigation. **Oleg Shcherbakov:** Writing – original draft, Investigation. **Astghik Ghazaryan:** Writing – review & editing, Data curation, Conceptualization. **Tigran Hayrapetyan:** Writing – review & editing, Validation, Investigation, Conceptualization. **Alexander Malkhasyan:** Validation, Investigation. **Hasmik Gevorgyan:** Writing – review & editing, Investigation. **Arseny Makarikov:** Writing – review & editing, Methodology, Investigation. **Svetlana Kornienko:** Writing – review & editing, Investigation. **Ahmad Daryani:** Writing – review & editing, Writing – original draft, Validation, Supervision, Methodology, Investigation, Data curation, Conceptualization.
